# The Impact of Income Inequality on Subjective Environmental Pollution: Individual Evidence from China

**DOI:** 10.3390/ijerph18158090

**Published:** 2021-07-30

**Authors:** Baoxi Li, De Xiao

**Affiliations:** 1 Research Center of Open Economy, Hubei University, Wuhan 430062, China; baoxili@stu.hubu.edu.cn; 2 School of Business, Hubei University, Wuhan 430062, China

**Keywords:** income inequality, relative deprivation in income, subjectively perceived environmental pollution, subjective well-being, media exposures

## Abstract

Numerous studies have investigated the relationship between income inequality and objective environmental pollution, but few focus on the nexus between income inequality and subjective environmental pollution (SEP). Using micro data from the Chinese General Society Survey (CGSS) in 2013 and official statistical data at the provincial level, this paper tests the impact of individual-level income inequality on subjective environmental pollution in China. The results show that individual-level income inequality has an inverted U-shape relationship with subjective environmental pollution, which indicates that increasing the income inequality at the individual level will first rise and then reduce their perceived subjective environmental pollution after reaching the peak. For about 84% of respondents, their subjective environmental pollution decreases with the increase of individual-level income inequality. Furthermore, the heterogeneity analyses show that the income inequality of urban residents and of the locals have an inverted U-shape effect on SEP, and the SEP of females and of individuals with positive environmental attitude are more sensitive to the effect of income inequality. Additionally, we find that subjective well-being plays a mediating role in the relation between income inequality and SEP. Individual income inequality decreases their self-reported well-being, and an increase in well-being has a negative effect on their subjectively perceived environmental quality. We also find non-television media exposures, such as newspaper, magazine, broadcasting, Internet, and mobile custom messages, will amplify the effect of individual-level income inequality on subjective environmental pollution.

## 1. Introduction

Since China’s reform and opening up in 1978, China has made great progress in economic development and poverty alleviation, but income inequality and environmental pollution issues followed. Income inequality and environmental pollution are very serious in China. Since 2000, income inequality in China has remained at a high level [1,2]. At the same time, environmental quality in China does not perform as well as economic development [3], especially air pollution and water pollution [4]. Therefore, it is necessary and urgent to research the tradeoff between income inequality and environmental pollution.

Existing papers have discussed the relationship between income inequality and environmental pollution [5,6,7,8,9], especially, the impact of income inequality on environmental pollution [10]. However, they mainly focused on the objective (actual) environmental pollution and ignored subjective (perceived) environmental pollution. The subjective environmental pollution, also called perceived environmental pollution [11], is measured by the respondents’ cognitive evaluation on environmental quality [12,13]. Subjective environmental pollution has made the public worried and uncertain about the future risks and threatens the public’s emotional and mental health [13]. Hence, the decrease in subjective environmental pollution has an important meaning for the public.

The reason why inequality matters is that it emphasizes the extent to which individuals feel relatively deprived—that is, the gap where one stands relative to others [14,15]. Income inequality has an important impact on socioeconomic development. An increase in income inequality reduces the growth of economy [16], destroys the stability of society [17,18], and crowds out social trust [19]. Especially, income inequality has a negative correlation with individual physical health [20] and self-reported health [21,22,23]. The impacts of income inequality on socioeconomic outcomes have a negative effect on social welfare [24] and reduce the happiness and life satisfaction of residents [19,25,26,27], which results in the decline of subjective well-being [28,29]. The existing literature indicates that perceived subjective environmental pollution is not only affected by objective environmental pollution [30,31,32,33] but also by sociodemographic characteristics [32,34,35] and psychological factors [32,36]. Those declaring themselves unhappy generally tend to have more negative evaluation on perceived environmental pollution [31]. Based on those, we infer that subjective well-being possibly impacts the relationship between income inequality and subjective environmental pollution. Income inequality has a negative effect on subjective well-being, and an increase in subjective well-being reduces individual perceived SEP. Additional, mass media increase people’s perceived environmental pollution and risk level [37]. Incomplete scientific information on the environment had the most impact on low-income households [38]. Hence, the nexus between income inequality and perceived environmental quality could be affected by mass media exposure.

To study the effect of individual income inequality on perceived subjective environmental pollution, we first measure individual income inequality; then, we combine individual information from CGSS with provincial socioeconomic and objective environment data from the National Bureau of Statistics of China (NBS) and Atmospheric Composition Analysis Group (ACAG). We use an ordered logistic regression model to identify the relationship and use other methods to test the robustness of the nexus. Finally, we discuss the possible mechanisms of individual income inequality on subjective environmental pollution from subjective well-being and media information exposure.

As a result, our main contributions for this paper are threefold. Firstly, from income deprivation, this paper analyzes, for the first time, the effect of income inequality on environmental pollution using individual-level data. Secondly, individuals’ feelings are given full consideration in this paper. This paper focuses not only on the public’s evaluation on environment but also on their feeling of relative deprivation in income. It assesses the relationship between income inequality and environmental pollution from the individual dimension and reveals how individuals with different levels of income inequality evaluate the environmental quality. Thirdly, this study, using China as the largest developing country, shows a nexus between individual income inequality and subjective environmental pollution and discusses the possible mechanisms of this effect. Income inequality and subjective environmental pollution impact individuals’ happiness and the sustainability of economic development. Hence, it is very important for China’s government to narrow income inequality and improve environmental quality.

Income inequality and environmental pollution have seriously threatened the sustainability of China’s development. Especially, subjective environmental pollution decreases the residents’ happiness and social welfare. As a result, for China’s government and policy makers, it is very important and meaningful to gauge to what extent and how subjective environmental pollution is affected by individual income inequality. Understanding the nexus between subjective environmental pollution and individual income inequality is vital to assess and implement the redistribution policy in income.

## 2. Literature Review

Considerable studies have showed that economic growth has an inverted U-shape effect on income inequality [39] and environmental pollution [40,41]. Based on that, some scholars investigated the relationship between income inequality and objective environment pollution from four folds.

A pioneering economist to study this topic is Boyce. He thought that the rich and powerful people benefited from environmental degradation and that the powerless and poor people bore the cost of environmental degradation. Therefore, he first proposed an “inequality hypothesis” that inequalities of power and wealth would lead to environmental degradation [42]. Based on the critical evaluation of the “inequality hypothesis”, Scruggs (1998) [43] proposed that the rich had higher environmental concerns and preferred less environmental degradation than the poor and that inequality would improve environmental quality. Some scholars attempted to use marginal propensity emission (MPE) to explain the effect of income inequality on the environment [44,45,46]. When MPE fell as income rose, that is, the poor tended to have a higher MPE than the rich, increasing income inequality would improve environmental quality. If MPE increased with rising income, that is, the rich tended to have a higher MPE than the poor, declining income inequality would reduce environmental pollution [45]. Finally, some scholars used the “Veblen effect” to explain the effect of income inequality on environment [44,46,47]. Due to the “Veblen effect”, work hours increased as income inequality rose [48]. Individuals in a given class preferred to compare themselves with the superior social class by emulating their consumption. Income inequality increased work hours and consumption, both of which would lead to environmental degradation [44,46,47,49].

Existing papers also examined the effect of income inequality on environmental pollution from experience. Some scholars found some cases in which income inequality measured by the Gini index deteriorated the environmental quality [50,51,52], improved the environmental quality [53], or had no impact on the environment [54] by using American data at the state level, Japanese data at the city level, or cross-country data. However, Chen et al., (2020), Baležentis et al., (2020), Grunewald et al., (2017), Jorgenson et al., (2017), and Uddin et al., (2020) [7,44,46,47,49] used data at the country level. They found that the income inequality measured by Gini index had a non-linear effect on carbon emission. This effect was correlated with time, income level, and GDP per capita. There are also abundant studies examining the effect of income inequality on the environment in China. Using inter-provincial data, the income gap measured by the Gini index increased carbon emission, and income inequality had a greater impact on carbon emission in eastern regions [55,56,57]. However, using data at the same level, some scholars found income inequality measured by the Gini index [5] and by the urban-rural income gap [58] had restricted the emission of pollutants and improved environmental quality.

In general, existing papers on the relationship between income inequality and the objective environment mainly employ macro data and cannot get consistent conclusions due to the differences among core indicators, the source, and the range of data. Until recently, Golley and Meng (2012) [59] used micro data from China’s Urban Household Income and Expenditure Survey in 2005 and used MPE to explain the nexus between income inequality and the environment. They found that the rich households emitted more CO_2_ both directly and indirectly than the poor households, and they thought that income redistribution from the rich to the poor would lead to the decline of income inequality and CO_2_ emission in tandem. Hence, Chiarini et al., (2020) [31] referred to Jorgenson et al., (2017) [46] and used MPE to explain the relation between income inequality and perceived subjective environmental pollution. They found that a negative correlation existed between income inequality measured by the Gini index and perceived environmental quality in European countries. Higher income inequality meant a lower perceived environmental quality. Another possible reason was that income inequality may cause the poor to “select” residential areas with lower environmental quality [9]. Those respondents with lower income contributed less pollution but suffered more from pollution and thus reported a higher perceived environmental exposure [35].

Overall, although there have been some studies on the impacts of income inequality on environmental pollution, they mainly focused on income inequality at the group level, such as the Gini index [43,55,56,59], urban–rural income gap [58] or the income group in various deciles [59]. These studies ignored the effect of income inequality at the individual level on environmental pollution. Additionally, the existing papers mainly investigated objective environmental issues—carbon emission, haze, etc. The studies in the literature on the nexus between individual income inequality and subjective environmental quality are relatively scare. In addition, existing papers mainly provided the experiences on the effect of income inequality on subjective environmental quality in European countries [31]. As such, this study fills the academic gap and contributes to the literature by quantitatively exploring, for the first time, the effect and mechanism of individual income inequality on subjective environmental pollution on the basis of China’s individual-level data.

## 3. Materials and Methodology

### 3.1. Data

The individual-level information, including subjective environmental pollution and socioeconomic characteristics, is collected from the 2013 waves of CGSS. CGSS is jointly conducted by Renmin University of China and the Hong Kong University of Science and Technology since 2003, covering rural and urban areas. CGSS 2013 covers 11438 observations in 28 mainland provincial administrative units (excluding Tibet, Xinjiang, Hainan, Taiwan, Hong Kong, and Macao). The final analysis sample includes 7314 observations after eliminating the missing values for the studied variables. We also use socioeconomic indicators and objective environmental pollution from the China Statistical Yearbook published by the NBS and ACAG in 2013 to control provincial characteristics.

#### 3.1.1. Subjective Environmental Pollution

The survey of CGSS 2013 contains 12 questions covering air, water, noise, garbage, vegetation, food, desertification, biodiversity, etc. to measure the respondents’ subjective evaluation on environmental quality. The scores of these relating variables from 1 to 7 stand for “very serious” to “no problem”, where 1 denotes “very serious”, 2 stands for “somewhat serious”, 3 is “not very serious”, 4 denotes “not serious”, 5 indicates “indifferent”, 6 denotes “no concern/unclear”, and 7 is “no problem”. To reflect environmental pollution more directly, we reassign the value and combine item 6 and item 7. We employ that 0 denotes “this problem does not exist”, 1 stands for “indifferent”, 2 indicates “not serious”, 3 is “not very serious”, 4 is “somewhat serious”, and 5 stands for “very serious”. The larger the value, the worse the subjective environmental quality. The first five questions—air pollution, water pollution, noise pollution, industrial waste pollution, and garbage pollution—measure pollution that affect human health and safety, and the remaining seven questions measure the severity of environmental degradation. Therefore, we mainly use the first five questions (How serious is the air pollution/water pollution/noise pollution/industrial waste pollution/garbage pollution problem in your area?) to measure subjective environmental pollution (SEP). The SEP is measured by the arithmetic mean of the five environmental variables.

#### 3.1.2. Income Inequality

Referring to [15,23,60], we employ relative deprivation in income (RD) to measure individuals’ income inequality. This paper employs the Yitzhaki index and Kakwani index to measure relative deprivation in income. The previous literature shows that the Yitzhaki index as follows: $Yitzhaki\;index_{i}=\frac{\sum \left(y_{j}-y_{i}\right)}{n}$ for all $y_{j}>y_{i}$, where $y_{i}$ is the income of individual *i* [61]. The index (Yitzhaki index is standardized, and a one-unit increase of Yitzhkai index means an increase of one standard deviation [14]. The Yitzhkai index is sensitive to the scale of income; it is not appropriate in panel data [62]. Considering that we use cross-sectional data, the challenge will not be taken into consideration) equals the sum of the difference of income between individual *i* and others who are higher than individual *i* and then divided by the size of reference group (*n*). We use the Kakwani index in our robustness test. The Kakwani index is the Yitzhaki index divided by the mean income of the reference group ($Kakwani\;index_{i}=\frac{\sum (y_{j}-y_{i})}{n\bar{y}}$, for all $y_{j}>y_{i}$, where $\bar{y}$ is the mean income of the reference group). Referring to Jones and Wildman (2008) [63], we use a national reference group. The larger the Yitzhaki index and Kakwani index of the individual *i*, the more unequal to individual *i*. The value of the Yitzhaki index is more than zero, and the range of the Kakwani index is from zero to one. (The mean of the Kakwani index for an individual in the reference group is equal to the Gini coefficient of the reference group [62], and the mean of the Yitzhaki index for individuals in the reference group is the mean income multiplied by the Gini coefficient of the reference group [14]).

Some househusbands and housewives have no income, but their spouses may have higher income, and some families have higher income because of the larger scale of family. Therefore, we use income inequality calculated by individual income (Individual income is related to the question, “What was your personal total income last year (2012)?”), household income (Household income is related to the question, “What was your family’s total annual income in 2012?”) and household income per capita (Household income per capita equals the total household income divided by household size that is measured by the number of family members), considering that different income only represents one aspect of the respondents in income inequality. Yitzhaki and Kakwani denote the Yitzhaki index and Kakwani index, respectively. ${in}_{i}$, ${in}_{h}$, and ${in}_{p}$ represent individual income, household income, and household income per capita, respectively. $r_{a}$ denotes that the reference group includes all respondents in our sample, and $r_{s}$ indicates that the reference group only includes individuals that have non-zero income. The larger the mean and standard error of the Yitzhaki index, the higher the volatility of the Yitzhaki index. We will take the logarithm of the Yitzhaki in our study to reduce heteroskedasticity. The Kakwani index has a relatively smaller mean and standard error; we will directly use Kakwani in our empirical model. We use the $Yitzhaki_{in_{i}r_{a}}$ index as the main measure of income inequality and the other seven indicators as alternative measures for robustness checks.

#### 3.1.3. Control Variables

This paper also controls environmental behavior (env behavior) (The behavior of environment is related to question, “Have you actively participated in complaints and appeals for environmental issues in the past year?”, and the response that 1 is often, 2 is occasionally, and 3 denotes always), environmental knowledge (env know) (The knowledge of environment is related to 10 items of the respondents’ grasp of knowledge on environmental protection. If the respondent answers right, he/she gets one score; otherwise, he/she gets zero score. Then, we sum the score of each item of each respondent and get the score of the respondent’s environmental knowledge), gender (gender), environmental attitude (env attitude) (Environmental attitude is a dummy variable, which is related to the questions, “What do you think is the most important problem to be solved in the following social issues?”. If the respondent places environmental issue as one of the first three problems needed to be solved, the value takes one; otherwise, it takes zero), cognitive ability (ind cog) (Individual cognitive ability is related to questions, “Which level do you think you have the listening ability in Mandarin/English?” and “Which level do you think you have the speaking ability in Mandarin/English?”. The score of the four questions is from 1 to 5, where 1 denotes that the respondent cannot understand by listening or cannot speak the language at all, and 5 means that the respondent is excellent in each ability. We take the mean value of the score of the four abilities), health (health) (Health is related the question, “What do you think is your status of current physical health?”. The score ranges from 1 to 5, and the higher the score, the better the status of physical health), well-being (well-being) (Well-being is related to the question, “In general, do you feel happy in your life?” The score ranges from 1 to 5, and the higher the score, the happier life), migrant or not (migrant), and household income per capita (lnphi) at the individual level, and the paper controls economic growth, population, and pollution at the provincial level. To reduce heteroskedasticity, we take the logarithm of household income per capita and the logarithm of control variables at the provincial level. Control variables at the provincial level include the logarithms of GDP per capita (lnPGDP) and its square term (lnPGDP2), population density (pop den), the concentration of PM_2.5_ (pm_2.5_), the quantity of household refuse (garbage), the emission of smoke (smoke), the emission of SO_2_ (so2), the emission of nitrogen oxides (nox), and the discharge of waste water (wat poll). PM_2.5_ data are from ACAG, and other control variables at the provincial level are from NBS. Table 1 displays the descriptive statistics of control variables.

### 3.2. Empirical Method

Since the dependent variable, subjective environmental pollution, is divided into point grades from 0 to 5, belonging to ordered qualitative variables, we mainly use the ordered logit model to analyze the impact of individual-level income inequality on subjective environmental pollution [64], and we employ an ordinary least square (OLS) and ordered probit model for a robustness check. Under the framework of EKC theory, Chen et al., (2020), Baležentis et al., (2020), Grunewald et al., (2017), Jorgenson et al., (2017), Uddin et al., (2020), and Wu and Xie (2020) [7,44,46,47,49,65] found that a non-linear nexus exists between income inequality and environment. Therefore, we set the basic model as follows:

(1) $$pollution_{i}=\alpha_{0}+\alpha_{1}inequality_{i}+\alpha_{2}inequality_{i}^{2}+X^{\prime }\gamma +\epsilon_{i}$$

where *i* is respondent *i*, ϵ denotes error term, α and γ are the estimation coefficients, and pollution measures the subjective environmental pollution. inequality represents individual income inequality, and $inequality^{2}$ denotes the square of individual income inequality. *X* represents the control variables, including individual characteristics and provincial factors. Therefore, the coefficients of interest, $\alpha_{1}\;\mathrm{and}\;\alpha_{2},$ denote the effect on individual-level income inequality on subjective environmental pollution. The signs of $\alpha_{1}\;\mathrm{and}\;\alpha_{2}$ show the shape between individual-level income inequality and subjective environmental pollution.

After determining the estimation methods, it is necessary to conduct diagnostic tests of the models. Firstly, we conduct tests of the Pearson correlation coefficient (see Table A1, Appendix A). The correlation coefficients are above 0.9 for some variables. Hence, there might be multicollinearity between dependent variables. It could decrease the statistical significance of our results. Secondly, we judge the number of explanatory variables by using information criteria (see Table A2). The model including individual characteristics and city factors is a better model. Thirdly, we conduct Ramsey RESET tests to test whether there exist omitted variables (see Table A3). The results show that there exist omitted variables in our three models, but the omitted information in the model that controls individual and city factors is fewer. Based on those, we conduct robustness tests to prove the robustness of the effect of income inequality on subjective environmental pollution. We mainly illustrate the results in the models that include both individual characteristics and city factors.

### 3.3. Mechanism Analysis

Referring to Baron and Kenny (1986) [66], we set models as follows and use a causal step approach to estimate the role of subjective well-being in the nexus between individual income inequality and subjective environmental pollution:

(2) $$pollution_{i}=\alpha_{0}+\alpha_{1}inequality_{i}+\alpha_{2}inequality_{i}^{2}+X_{1}^{'}\gamma_{1}+\epsilon_{1i}$$

(3) $$well-being_{i}=\beta_{0}+\beta_{1}inequality_{i}+X_{2}^{'}+\epsilon_{2i}$$

(4) $$pollution_{i}=\alpha_{0}^{'}+\alpha_{1}^{'}inequality_{i}+\alpha_{2}^{'}inequality_{i}^{2}+\alpha_{3}^{'}\left(well-being_{i}\right)+X_{1}^{'}\gamma_{1}+\epsilon_{3i}$$

where $\alpha_{1}$ and $\alpha_{2}$ in Equation (2) denote the total effect of income inequality on subjective environmental pollution, $\beta_{1}$ denotes the effect of income inequality on subjective well-being, and $\alpha_{1}^{'}$ and $\alpha_{2}^{'}$ denotes the direct effect of income inequality on subjective environmental pollution. The indirect effect of subjective well-being on subjective environmental pollution is the product of $\beta_{1}\;\mathrm{and}\;\alpha_{3}^{'}$. If $\beta_{1}\;\mathrm{and}\;\alpha_{3}^{'}$ are statistically significant, it means that subjective well-being plays a mediating role in the relationship between income inequality and subjective environmental pollution. X is the control variables, and *ε* denotes error term. The subscript, Arabic numerals, differentiates the differences in each model.

In general, the individuals that suffer from more income inequality use less media than others (see Table A4). Meanwhile, the individuals exposed to more media have more awareness and behavior of environmental pollution [37,67]. It might raise a question of which mass media exposure can play a moderating role in the effect of income inequality on subjective environmental pollution. Hence, we refer to Baron and Kenny (1986) [66] to construct Equation (5) and discuss the effect of the mass media exposure on the nexus between income inequality and subjective environmental pollution.

(5) $$pollution_{i}=\alpha_{0}+\alpha_{1}inequality_{i}+\alpha_{2}inequality_{i}^{2}+\alpha_{3}media_{i}+\alpha_{4}inequality_{i}*media_{i}+X^{\prime }\gamma +\epsilon_{i}$$

where $\alpha_{4}$ denotes the effect of income inequality on subjective environmental pollution as the change of media exposures, and other variables have the same definition as above. Media are related to question, “In the past year, your usage of the following media is”, where 1 denotes never, 2 is rarely, 3 presents sometimes, 4 is often, and 5 means very regularly. Media include magazines, newspapers, broadcasting, television, Internet (including surfing the Internet with a mobile phone), and mobile custom messages.

## 4. Results

### 4.1. Baseline Results

We estimate the effect of income inequality on SEP by using Equation (1) and present the results in Table 2. The results in columns (1)–(3) are estimated using the OLS model, and columns (4)–(6) are the estimation results of an ordered logit model.

The linear and quadratic coefficients of inequality are significantly positive and negative at the 1% level, respectively, showing an inverted U-shape relationship between income inequality and subjective environmental quality. The turning point of income inequality is around 8.4, which indicates that the subjective environmental pollution of approximately 84% of respondents increases with the decrease of individual-level income inequality.

In conclusion, there exists an inverted U-shape relationship between individual-level income inequality and subjective environmental pollution, among which approximate 84% of respondents’ subjective environmental pollution decreases with the increase of income inequality. With the increase of income inequality at an individual level, subjective evaluation on environmental pollution is first getting worse and then getting better after reaching the peak.

### 4.2. Robustness Test

#### 4.2.1. Regression for Different Subjective Environmental Pollution

We use the five subjective environmental pollution indicators: air pollution, water pollution, noise pollution, industrial waste pollution, and garbage pollution, as dependent variables to analyze the effect of individual-level income inequality on different subjective environmental pollution. The results are reported in Table 3.

Similar to the results in Table 2, the linear coefficients of inequality (inequality) are significantly positive at the 10% level and the quadratic coefficients of inequality (inequality^2^) are negative at the 5% significance level, which support there being an inverted U-shape relation between income inequality at an individual level and subjective environmental pollution. Similar to subjective environmental pollution, the turning point of income inequality for subjective air/water/noise/industrial waste pollution is around 8.5, but the turning point for subjective garbage pollution is larger than 8.5. The results show for over 84% of respondents that their subjective air, water, noise, and industrial waste pollution are negatively correlated with income inequality, but that only 38.8% of respondents’ subjective garbage pollution decreases following the increase of income inequality. The signs of coefficients of the control variables are the same as the results in Table 2, which shows that the estimation results are robust.

Therefore, individual-level income inequality has a robust inverted U-shape effect on subjective environmental pollution.

#### 4.2.2. Regression for Other Income Inequality Indicators

This part measures income inequality employing other methods to test the robustness of the effect of individual-level income inequality on subjective environmental pollution. The results are reported in Table 4. The coefficients of linear inequality (inequality) are positive, and the coefficients of quadratic inequality (inequality^2^) are significantly negative. It supports that an inverted U-shape relationship exists between income inequality and subjective environmental pollution.

#### 4.2.3. Regression for Other Methods

Considering that heteroscedasticity possibly exists among different groups, we report the clustered robust standard errors in columns (1)–(3) in Table 5. The standard error in columns (1)–(3) are clustered at province, city, and county levels, respectively. Additionally, the results in column 4 in Table 5 are estimated by ordered probit model. The results on inequality (inequality) and its quadratic term (inequality^2^) are similar to the baseline results. It supports that an inverted U-shape relationship exists between individual-level income inequality and subjective environmental pollution.

Consequently, individual-level income inequality has a robust inverted U-shape effect on subjective environmental pollution. With the increase of income inequality, subjective environmental pollution is first getting worse and then getting better.

## 5. Discussion

Increasing individual-level income inequality first increases and then decreases subjective environmental pollution. However, there also exist heterogenous effects of individual-level income inequality on subjective environmental pollution, due to the differences of income inequality and subjective environmental pollution among various groups. In the following analysis, we will discuss the different effects of income inequality on subjective environmental pollution in various contexts.

### 5.1. Difference between the Urban and Rural Residents

According to the living places of the respondents, the samples are divided into the urban area group and rural area group (If the respondents live in a rural area, they are classified into the rural area group; otherwise, they are in the urban area group. The urban area group includes the respondents living in the central area, suburban fringe, rural–urban fringe zone of the city or county, and in the town outside of the city or county). The respondents living in the rural area have higher income inequality and have better evaluations on environmental quality than those living in the urban area (see Table 6). Further investigation finds that the coefficients of linear inequality (inequality) and its quadratic term (inequality^2^) are not significant in the rural area (in columns 4–6 in Table 7), but that the coefficients of linear (inequality) and square inequality (inequality^2^) in the urban area are significantly negative and positive at the 1% level (see columns 1–3 in Table 7), respectively. It indicates that individual-level income inequality has an inverted U-shape effect on subjective environmental pollution in the urban area group, among which approximately 71% of respondents’ subjective environmental pollution decreases following the increase of income inequality.

Therefore, individual-level income inequality has an inverted U-shape relationship with subjective environmental pollution for the urban residents and has little impact on the subjective environmental pollution for the rural residents.

### 5.2. The Differences between the Local and Migrants

We define a migrant as one whose hukou is not the same place as their habitation or he/she changed the registered address of their hukou (If the hukou of the respondent is outside of the district, county, and county-level city, the respondent migrates to the locality, or the hukou is not in the locality but he/she lives in the locality, we define these respondents as migrants; otherwise, we define them as the local people). Based on that, the samples are classified into locals and migrants. Migrants have lower income inequality and have worse evaluation on environment quality compared with the locals (see Table 8). We further report the estimation results in Table 9. The coefficients of linear inequality (inequality) are positive, and those of its square term (inequality^2^) are negative. The results of the locals are significant at the 1% level but those of migrants are not significant at the 10% level. Therefore, in the local group, there exists an inverted U-shape relationship between individual-level income inequality and subjective environmental pollution. According to the calculation, for about 95.4% of local people, their subjective environmental pollution decreases with the increase of individual income inequality.

Therefore, we can conclude that individual-level income inequality first increases and then decreases subjective environmental pollution for the locals. Meanwhile, the individual-level income inequality of migrants has little impact on subjective environmental pollution when considering the effect of other factors.

### 5.3. Difference in Gender

We classified the samples into female and male groups according to their gender. Women significantly suffer from more income inequality than men, but there are no significant differences in subjective environmental pollution between males and females (see Table 10). The coefficients of linear inequality (inequality) and those of its square term (inequality^2^) are positive and negative at the 5% significance level, respectively (see Table 11), which means that an inverted U-shape curve exists between individual income inequality and subjective environmental pollution in the two groups. However, the coefficients of individual-level income inequality in the female group are larger. It means that females are more sensitive to the effect of individual income inequality on subjective environmental pollution.

Therefore, we conclude that individual-level income inequality has an inverted U-shape effect on subjective environmental pollution in the two groups. Females are more sensitive for the effect of individual-level income inequality on environmental pollution.

### 5.4. Difference in Attention on Environmental Issues

We divide the samples into the positive environmental attitude group and negative environmental attitude group according to whether the respondents put environmental issues as one of the first three most urgent problems to be solved (if the respondents put environmental issues as one of the first three most urgent problems that need to be solved, we think that they have a positive environmental attitude; otherwise, they are in the negative environmental attitude group). The respondents in the negative environmental attitude group have higher income inequality and better evaluation on environmental quality (see Table 12). The coefficients of inequality (inequality) and those of its square term (inequality^2^) are significantly negative and positive, respectively, in the two groups (see Table 13), which indicates that there exists an inverted U-shape relationship between individual income inequality and subjective environmental pollution. The coefficients of inequality (inequality) and those of its quadratic term (inequality^2^) in the positive environmental attitude group are larger, indicating that the subjective environmental pollution of the respondents in the positive environmental attitude group is more sensitive to the effect of individual-level income inequality.

In conclusion, individual-level income inequality has an inverted U-shape effect on subjective environmental pollution in the two groups, and the respondents in the positive group have a more sensitive reaction to individual-level income inequality.

### 5.5. Mechanism

#### 5.5.1. The Effect of Subjective Well-Being

Using Equations (2)–(4), we estimate the role of subjective well-being in the effect of individual income inequality on subjective environmental pollution. The estimation results are shown in Table 14. The coefficients of inequality (inequality) and those of its square term (inequality^2^) are significantly positive and negative, respectively (in columns 1, 3, 4, and 6 in Table 14), which means that individual-level income inequality has an inverted U-shape effect on subjective environmental pollution. The coefficients of inequality to subjective well-being are negative (in columns 2 and 5 in Table 14), which indicates that income inequality decreases perceived well-being of individuals. The coefficients of subjective well-being to subjective environmental pollution are significantly negative at a 1% level (in columns 3 and 6 in Table 14), which means that an increase in subjective well-being decreases individual perceived subjective environmental pollution.

Therefore, income inequality increases the subjective environmental pollution via subjective well-being. Income inequality decreases the individual subjective well-being, and a decrease in subjective well-being increases perceived environmental pollution.

#### 5.5.2. The Effect of Media Exposures

Using Equation (5), we estimate the effect of media exposure on the nexus between individual income inequality and subjective environmental pollution. The results are reported in Table 15. Similar to the results in the previous section, the results in Table 15 support that an inverted U-shape relation exists between individual-level income inequality and subjective environmental pollution. The coefficients of the interactions of inequality and non-television media are significantly positive, which indicates that the effect of individual-level income inequality on subjective environmental pollution increases alongside an increase in non-television media exposure. Interestingly, the coefficient of the interaction of inequality and television media is negative but not significant, which supports the conclusion proposed by Lu and Sun (2018) [68] that television media has no significant impacts on environmental knowledge and environmental preference. The possible reasons are that modern television has limited abilities in the dissemination of environmental knowledge and environmental governance and that the environmental preference of the public is affected by various types of subjective information from government officials, experts, and environmental organizations through television.

In conclusion, increasing the frequency of respondents’ access to non-television media will amplify the effect of individual-level income inequality on subjective environmental pollution. Television media plays no role in the effect of income inequality on subjective environmental pollution.

## 6. Conclusions

This paper uses CGSS2013 data and an ordered logit model to analyze the relationship between individual-level income inequality and subjective environmental pollution. The results show that individual-level income inequality has an inverted U-shape effect on subjective environmental pollution; that is, the increase of an individual’s income inequality will first increase then decrease the respondents’ feeling of environmental pollution after reaching the peak. The subjective environmental pollution increases following the decrease of income inequality for approximately 84% of respondents in China. The robustness test supports these conclusions.

Further classifying the samples according to the characteristics of respondents, we find existing different effects of individual-level income inequality on subjective environmental pollution among different groups. Income inequality has an obviously inverted U-shape effect on subjective environmental pollution for the urban residents and the locals, but we cannot get an inverted U-shape curve relation between income inequality and subjective environmental pollution for the rural residents and the migrants. An inverted U-shape curve exists between individual-level income inequality and subjective environmental pollution under the classifications of gender and environmental attitude. However, for females and the respondents who have positive environmental attitudes, the subjective environmental pollution is more sensitive to the effect of income inequality. We also find that subjective well-being plays a mediating role in the effect of income inequality on subjective environmental pollution. Income inequality increases the perceived subjective environmental pollution via subjective well-being. The results show that exposing to non-television media plays a moderation role in the effect of income inequality on subjective environmental pollution, but the moderation effect of television media is not significant. The effect of income inequality on subjective environmental pollution increases as non-television media exposure rises.

These findings enhanced our understanding of the relationship and potential mechanism between individual income inequality and subjective environmental pollution. It can be seen that individual income inequality has an inverted U-shape effect on subjective environmental pollution. We strongly recommend that the government implement prudent income redistribution policies to improve individuals’ subjective environmental quality. Narrowing the income gap among individuals does not always improve their subjective evaluation on environmental quality. When an individual-level income gap is larger, income redistribution from the rich to the poor will increase the subjective evaluation of environmental pollution of the overall residents. When the income gap among individuals is smaller, narrowing the income gap among individuals will decrease the subjective environmental pollution of residents. Hence, when individual income inequality is low, income distribution from rich to poor may decrease subjective environmental pollution; when individual income inequality is high, the income distribution policy implemented by the government plays no roles in improving individuals’ subjective environmental quality. Additionally, subjective environmental pollution is mainly decided by objective environmental pollution. The government should positively improve objective (actual) environmental pollution to decrease the residents’ subjective environmental pollution.

There still exist some limitations in the paper. First, due to the limitation of data, this paper only controls air pollution, water pollution, and garbage pollution at the province level; meanwhile, there may exist other pollutions that impact subjective environmental pollution, such as waste pollution and so on. Hence, it is very necessary to use more micro data, such as city-level or county-level data, and more kinds of pollutants, to control the effect of objective environmental pollution in the future. Second, for protecting the privacy of the respondents, the CGSS team only releases the residential addresses of the respondents at the province level, so we cannot merge more detailed information at the street, county, or city level. The control variables at the province level may be too rough to control more factors that the respondents face. Based on that, using more micro and detailed information of each individual is another future direction. Third, we do not explain and verify the reason why an inverse U-shape relationship exists between individual-level income inequality and subjective environmental pollution, because of the lack of the habitations of the respondents. We infer the possible explanation that the poorer who suffer from more income inequality live in undeveloped areas with good environmental quality and the richer who have relatively less income inequality can freely choose the area with good environmental quality to live in; hence, they both have higher evaluations on environmental quality. Hence, future studies could use more detailed data to explain and verify the reason why individual income inequality affect subjective environmental pollution.

## Figures and Tables

**Table 1:** Descriptive statistics of variables.

	Obs.	Mean	sd	Min	Max
Air pollution	7314	2.74	1.64	0.000	5.000
Water pollution	7314	2.65	1.63	0.000	5.000
Noise pollution	7314	2.44	1.64	0.000	5.000
Industrial pollution	7314	2.10	1.72	0.000	5.000
Garbage pollution	7314	2.66	1.57	0.000	5.000
SEP	7314	2.52	1.30	0.000	5.000
$Yitzhaki_{in_{i}r_{a}}$	7314	12,761.41	6936.68	82.983	23,814.436
$Yitzhaki_{in_{i}r_{s}}$	6556	12,965.74	6952.87	93.695	26,808.352
$Yitzhaki_{in_{h}r_{s}}$	6835	27,689.21	13,874.85	0.000	58,454.809
$Yitzhaki_{in_{p}r_{s}}$	6835	10,386.36	4551.01	0.000	19,786.146
${Kakwani}_{{in}_{i}r_{a}}$	7314	0.54	0.29	0.003	1.000
${Kakwani}_{{in}_{i}r_{s}}$	6556	0.48	0.26	0.003	0.997
${Kakwani}_{{in}_{h}r_{s}}$	6835	0.47	0.24	0.000	0.992
${Kakwani}_{{in}_{p}r_{s}}$	6835	0.52	0.23	0.000	0.992
env behavior	7308	1.11	0.35	1.000	3.000
env know	7314	5.30	2.70	0.000	10.000
gender	7314	0.54	0.50	0.000	1.000
env attitude	7314	0.30	0.73	0.000	3.000
ind cong	7314	2.56	0.71	1.000	5.000
health	7313	3.82	1.03	1.000	5.000
well-being	7314	3.80	0.81	1.000	5.000
migrant	7310	0.28	0.45	0.000	1.000
lnphi	6835	9.43	1.02	5.116	16.118
lnPGDP	7314	10.78	0.42	10.050	11.514
lnPGDP2	7314	116.32	9.10	100.998	132.572
pop den	7314	7.89	0.41	6.965	8.620
pm25	7314	3.71	0.45	2.537	4.426
garbage	7314	6.34	0.58	4.305	7.646
nox	7314	4.18	0.59	2.582	5.107
so2	7314	4.04	0.71	2.163	5.103
wat poll	7314	12.29	0.66	9.997	13.668
smoke	7314	3.49	0.77	1.780	4.878

Note: the unit of Yitzhaki is Yuan, pop den, pm25, garbage, nox, so2, wat poll, and smoke are taken as a logarithm.

**Table 2:** The effect of individual-level income inequality on subjective environmental pollution: baseline results.

	(1)	(2)	(3)	(4)	(5)	(6)
	OLS			Ordered logit model		
inequality	1.546 ***	1.328 ***	0.886 ***	2.036 ***	1.904 ***	1.317 ***
	(5.65)	(4.66)	(3.16)	(5.29)	(4.68)	(3.20)
inequality^2^	−0.109 ***	−0.079 ***	−0.053 ***	−0.144 ***	−0.113 ***	−0.077 ***
	(−7.00)	(−4.83)	(−3.27)	(−6.58)	(−4.83)	(−3.25)
lnphi		0.176 ***	0.110 ***		0.259 ***	0.165 ***
		(7.46)	(4.73)		(7.60)	(4.85)
env behavior		0.296 ***	0.286 ***		0.439 ***	0.451 ***
		(6.96)	(6.78)		(6.71)	(6.62)
env know		0.048 ***	0.049 ***		0.063 ***	0.070 ***
		(7.36)	(7.70)		(6.69)	(7.49)
gender		−0.080 **	−0.061 *		−0.110 **	−0.078 *
		(−2.44)	(−1.92)		(−2.36)	(−1.69)
env attitude		0.074 ***	0.065 ***		0.093 ***	0.087 ***
		(3.89)	(3.40)		(3.40)	(3.07)
ind cong		0.191 ***	0.147 ***		0.278 ***	0.223 ***
		(7.61)	(5.91)		(7.54)	(5.97)
health		−0.007	−0.003		−0.008	−0.005
		(−0.43)	(−0.18)		(−0.38)	(−0.23)
well-being		−0.132 ***	−0.111 ***		−0.194 ***	−0.168 ***
		(−6.83)	(−5.82)		(−6.90)	(−5.85)
lnPGDP			−4.924 *			−6.250
			(−1.66)			(−1.47)
lnPGDP2			0.248 *			0.316
			(1.79)			(1.59)
pop den			0.034			0.055
			(0.82)			(0.90)
pm25			0.394 ***			0.573 ***
			(8.68)			(8.63)
garbage			0.562 ***			0.833 ***
			(8.79)			(8.79)
nox			−0.368 ***			−0.463 ***
			(−3.18)			(−2.76)
so2			0.255 ***			0.305 ***
			(3.79)			(3.05)
wat poll			−0.666 ***			−0.990 ***
			(−10.07)			(−10.39)
smoke			0.150 *			0.186
			(1.87)			(1.55)
N	7314	6825	6825	7314	6825	6825

Notes: The parentheses show *t* value of robust standard error. Asterisks indicate the statistical significance, where *, **, and *** denote significance at 10%, 5%, and 1% levels, respectively.

**Table 3:** The effect of individual-level income inequality on different subjective environmental pollution.

	(1)	(2)	(3)	(4)	(5)
	Air pollution	Water pollution	Noise pollution	Industrial waste pollution	Garbage pollution
inequality	1.252 ***	0.945 **	1.973 ***	0.752 *	0.819 *
	(2.79)	(2.32)	(4.77)	(1.71)	(1.90)
inequality^2^	−0.074 ***	−0.056 **	−0.118 ***	−0.044 *	−0.043 *
	(−2.86)	(−2.40)	(−4.95)	(−1.75)	(−1.72)
Individual factors fixed effect	Yes	Yes	Yes	Yes	Yes
Provincial factors fixed effect	Yes	Yes	Yes	Yes	Yes
N	6825	6825	6825	6825	6825

Notes: The parentheses show the *t* value of robust standard error. Asterisks indicate the statistical significance, where *, **, and *** denote significance at the 10%, 5%, and 1% levels, respectively. The results are estimated by an ordered logit model.

**Table 4:** The effect of individual-level income inequality on subjective environmental pollution: robustness test based on different measurements of income inequality at in individual level.

	(1)	(2)	(3)	(4)	(5)	(6)	(7)
	$Yitzhaki_{in_{i}r_{s}}$	$Yitzhaki_{in_{h}r_{s}}$	$Yitzhaki_{in_{p}r_{s}}$	${Kakwani}_{{in}_{i}r_{a}}$	${Kakwani}_{{in}_{i}r_{s}}$	${Kakwani}_{{in}_{hi}r_{s}}$	${Kakwani}_{{in}_{p}r_{s}}$
inequality	1.784 ***	1.835 **	6.231 ***	0.435	1.400 ***	1.119 ***	0.121
	(3.84)	(2.08)	(4.38)	(1.13)	(3.35)	(2.59)	(0.21)
inequality^2^	−0.103 ***	−0.086 *	−0.362 ***	−0.597 *	−1.780 ***	−0.910 **	−1.601 ***
	(−3.84)	(−1.86)	(−4.27)	(−1.91)	(−4.75)	(−2.34)	(−3.63)
Individual factors fixed effect	Yes	Yes	Yes	Yes	Yes	Yes	Yes
Provincial factors fixed effect	Yes	Yes	Yes	Yes	Yes	Yes	Yes
N	6211	6825	6825	6826	6212	6826	6826

Notes: Asterisks indicate the statistical significance, where ***, **, and * represent significance at 1%, 5%, and 10% levels, respectively. The parentheses show the *t* value of robust standard error. The results in this table are estimated by an ordered logit model. N is the number of observations.

**Table 5:** The effect of individual-level income inequality on subjective environmental pollution: robustness test based on cluster analysis and ordered probit model.

	(1)	(2)	(3)	(4)
	clustered at province level	clustered at city level	clustered at county level	probit
inequality	1.317 **	1.317 **	1.317 **	0.737 ***
	(2.39)	(2.46)	(2.53)	(3.11)
inequality^2^	−0.077 **	−0.077 **	−0.077 ***	−0.043 ***
	(−2.36)	(−2.41)	(−2.59)	(−3.17)
Individual factors fixed effect	Yes	Yes	Yes	Yes
Provincial factors fixed effect	Yes	Yes	Yes	Yes
N	6825	6825	6825	6825

Note: The parentheses show *t* statistics, where columns 1–3 are *t* values of clustering robust standard error. Asterisks indicates the statistical significance, where *** and ** represent significance at 1% and 5% levels, respectively. Column 1 indicates clusters at the province level, column 2 indicates clusters at the city level, column 3 indicates clusters at the county level. Column 4 is estimated by an ordered probit model. N is the number of observations.

**Table 6:** *t* test of individual-level income inequality and subjective environmental pollution: differences in the urban and rural area.

	The Urban Area		The Rural Area		Diff.	*t*
	Mean	Obs.	Mean	Obs.		
inequality	9.061	4761	9.606	2550	−0.545 ***	−32.936
inequality^2^	82.659	4761	92.545	2550	−9.886 ***	−33.990
SEP	3.146	4761	2.364	2551	0.782 ***	25.280

Note: Diff. denotes the differences of mean value between the urban area group and rural area group, *t* is *t* statistics. Asterisks indicate the statistical significance, where *** denotes significance at the 1% level.

**Table 7:** The effect of individual-level income inequality on subjective environmental pollution: differences in the urban and rural area.

	(1)	(2)	(3)	(4)	(5)	(6)
	The urban area			The rural area		
inequality	0.982 **	1.219 ***	0.931 **	−1.029	−1.330	−1.329
	(2.43)	(2.93)	(2.24)	(−1.27)	(−1.58)	(−1.31)
inequality^2^	−0.069 ***	−0.071 ***	−0.053 **	0.040	0.067	0.068
	(−2.94)	(−2.90)	(−2.18)	(0.90)	(1.41)	(1.20)
Individual factors fixed effect	No	Yes	Yes	No	Yes	Yes
Provincial factors fixed effect	No	No	Yes	No	No	Yes
N	4761	4457	4457	2550	2368	2368

Notes: The parentheses show *t* value of robust standard error. Asterisks indicate the statistical significance, where ** and *** denote significance at the 5% and 1% levels, respectively. The results in this table are estimated by an ordered logit model. N is the number of observations.

**Table 8:** *t* test of individual-level income inequality and subjective environmental pollution: differences between the locals and migrants.

	Locals		Migrants		Diff.	*t*
	Mean	Obs.	Mean	Obs.		
inequality	9.332	5241	9.047	2069	0.285 ***	15.453
inequality^2^	87.545	5241	82.462	2069	5.083 ***	15.601
SEP	2.827	5242	2.990	2069	−0.163 ***	−4.793

Note: Diff. denotes the differences of mean value between the locals and migrants. *t* is *t* statistics. Asterisks indicate the statistical significance, where *** is significant at a 1% level.

**Table 9:** The effect of individual-level income inequality on subjective environmental pollution: differences between the locals and migrants.

	(1)	(2)	(3)	(4)	(5)	(6)
	Local			Migrant		
inequality	2.127 ***	2.004 ***	1.407 **	1.312 **	0.943	0.393
	(4.40)	(3.80)	(2.49)	(2.05)	(1.44)	(0.63)
inequality^2^	−0.152 ***	−0.124 ***	−0.087 ***	−0.094 **	−0.048	−0.015
	(−5.55)	(−4.12)	(−2.71)	(−2.54)	(−1.25)	(−0.40)
Individual factors fixed effect	No	Yes	Yes	No	Yes	Yes
Provincial factors fixed effect	No	No	Yes	No	No	Yes
N	5241	4885	4885	2069	1940	1940

Notes: The parentheses show *t* value of robust standard error. Asterisks indicate the statistical significance, where ** and *** denote significance at the 5% and 1% levels, respectively. The results in this table are estimated by an ordered logit model. N is the number of observations.

**Table 10:** *t* test of individual-level income inequality and subjective environmental pollution: differences in gender.

	The Female Group		The Male Group		Diff.	*t*
	Mean	Obs.	Mean	Obs.		
inequality	9.438	3372	9.091	3942	0.347 ***	21.094
inequality^2^	89.483	3372	83.210	3942	6.273 ***	21.612
SEP	2.888	3372	2.860	3943	0.029	0.937

Note: Diff. denotes the differences of mean value between the female group and male group, *t* is *t* statistics. Asterisks indicate the statistical significance, where *** denotes significance at the 1% level.

**Table 11:** The effect of individual-level income inequality on subjective environmental pollution: differences in gender.

	(1)	(2)	(3)	(4)	(5)	(6)
	The female group			The male group		
inequality	2.958 ***	2.700 ***	1.977 **	2.023 ***	1.763 ***	1.237 ***
	(3.71)	(3.02)	(2.26)	(4.48)	(3.87)	(2.59)
inequality^2^	−0.195 ***	−0.155 ***	−0.111 **	−0.148 ***	−0.106 ***	−0.074 ***
	(−4.40)	(−3.13)	(−2.28)	(−5.64)	(−3.92)	(−2.61)
Individual factors fixed effect	No	Yes	Yes	No	Yes	Yes
Provincial factors fixed effect	No	No	Yes	No	No	Yes
N	3372	3124	3124	3942	3704	3704

Notes: The parentheses show *t* value of robust standard error. Asterisks indicate the statistical significance, where **and *** denote significance at 5% and 1% levels, respectively. About 84.1% of females think that individual-level income inequality decreases subjective environmental pollution, and about 86.3% of males think that individual-level income inequality decreases subjective environmental pollution. N is the number of observations.

**Table 12:** *t* test of individual-level income inequality and subjective environmental pollution: differences in environmental attitude.

Variables	Negative Environmental Attitude		Positive Environmental Pollution		Diff.	*t*
	Mean	Obs.	Mean	Obs.		
inequality	9.292	6105	9.046	1209	0.246 ***	10.898
inequality^2^	86.826	6105	82.442	1209	4.384 ***	11.002
SEP	2.832	6106	3.080	1209	−0.248 ***	−6.021

Note: Diff. denotes the differences of mean value between the negative environmental attitude group and positive environmental attitude group, *t* is *t* statistics. Asterisks indicate the statistical significance, where *** denotes significance at 1% level.

**Table 13:** The effect of individual-level income inequality on subjective environmental pollution: differences in environmental attitude.

	(1)	(2)	(3)	(4)	(5)	(6)
	Negative attitude group			Positive attitude group		
inequality	1.872 ***	1.588 ***	0.858 *	2.312 ***	2.447 ***	2.143 ***
	(3.90)	(3.10)	(1.67)	(3.51)	(3.62)	(3.11)
inequality^2^	−0.134 ***	−0.095 ***	−0.051 *	−0.158 ***	−0.146 ***	−0.127 ***
	(−4.95)	(−3.26)	(−1.73)	(−4.07)	(−3.61)	(−3.06)
Individual factors fixed effect	No	Yes	Yes	No	Yes	Yes
Provincial factors fixed effect	No	No	Yes	No	No	Yes
N	6105	5696	5696	1209	1132	1132

Notes: The parentheses show *t* values of robust standard error. Asterisks indicate the statistical significance, where * and *** denote significance at 10% and 1% levels, respectively. The results in this table are estimated by an ordered logit model. About 86.8% of individuals in the negative group think that individual-level income inequality decreases subjective environmental pollution, and 78.2% of respondents in the positive group have the same opinion. N is the number of observations.

**Table 14:** The mechanism of the effect of individual-level income inequality on subjective environmental pollution: the effect of subjective well-being.

	(1)	(2)	(3)	(4)	(5)	(6)
	SEP	Well-being	SEP	SEP	Well-being	SEP
inequality	1.601 ***	−0.146 ***	1.588 ***	1.041 **	−0.193 ***	1.044 **
	(3.87)	(−3.77)	(3.91)	(2.47)	(−4.65)	(2.52)
inequality^2^	−0.092 ***		−0.092 ***	−0.059 **		−0.059 **
	(−3.88)		(−3.92)	(−2.43)		(−2.48)
Well-being			−0.160 ***			−0.138 ***
			(−5.73)			(−4.90)
Individual factors fixed effect	Yes	Yes	Yes	Yes	Yes	Yes
Provincial factors fixed effect	No	No	No	Yes	Yes	Yes
N	6825	7303	6825	6825	7303	6825

Notes: The parentheses show *t* values of robust standard error. Asterisks indicate the statistical significance, where ** and *** denote significance at 5% and 1% levels, respectively. The results in this table are estimated by ordered logit model. N is the number of observations.

**Table 15:** The mechanism of the effect of individual-level income inequality on subjective environmental pollution: the effect of media exposure.

	(1)	(2)	(3)	(4)	(5)	(6)
	newspaper	magazine	broadcasting	television	Internet (including surfing the Internet with mobile phone)	mobile custom message
inequality	0.590 **	0.631 **	0.875 ***	0.900 ***	0.457	0.610 **
	(2.01)	(2.23)	(2.97)	(3.28)	(1.48)	(2.22)
inequality^2^	−0.039 **	−0.043 ***	−0.054 ***	−0.052 ***	−0.034 **	−0.042 ***
	(−2.42)	(−2.73)	(−3.27)	(−3.31)	(−2.03)	(−2.70)
media	−0.234	−0.371 **	−0.230	0.004	−0.308 **	−0.407 ***
	(−1.48)	(−2.15)	(−1.42)	(0.02)	(−2.33)	(−2.86)
Inequality∗media	0.029 *	0.042 **	0.030 *	−0.002	0.036 **	0.049 ***
	(1.68)	(2.18)	(1.68)	(−0.09)	(2.50)	(3.07)
Individual factor fixed effect	Yes	Yes	Yes	Yes	Yes	Yes
Province factor fixed effect	Yes	Yes	Yes	Yes	Yes	Yes
N	6824	6822	6817	6819	6814	6818
R^2^	0.117	0.117	0.099	0.116	0.117	0.118

Notes: The parentheses show *t* values of robust standard error. Asterisks indicate the statistical significance, where *, **, and *** denote significance at 10%, 5%, and 1% levels, respectively. The results in this table are estimated by OLS. N is the number of observations.

## Data Availability

The data presented in this study are available through the website of CGSS (cnsda.ruc.edu.cn) (accessed on 30 September 2019).

## Appendix A

**Table A1:** Person correlation coefficient test.

	SEP	Inequality	Inequality^2^	Env Behavior	Env Know	Gender	Env Attitude	Ind Cong	Health	Well-Being	Migrant	Lnphi	lnPGDP	lnPGDP2	Pop Den	pm25	Garbarge	Nox	so2	Wat Poll	Smoke
SEP	1.000																				
inequality	−0.209 **	1.000																			
inequality^2^	−0.214 **	0.997 **	1.000																		
env behavior	0.122 **	−0.117 **	−0.117 **	1.000																	
env know	0.212 **	−0.310 **	−0.314 **	0.085 **	1.000																
gender	−0.009	−0.239 **	−0.245 **	0.025 *	0.093 **	1.000															
env attitude	0.083 **	−0.116 **	−0.117 **	0.040 **	0.109 **	−0.021	1.000														
ind cong	0.225 **	−0.364 **	−0.365 **	0.109 **	0.422 **	−0.025 *	0.100 **	1.000													
health	0.067 **	−0.178 **	−0.182 **	0.042 **	0.168 **	0.035 **	0.057 **	0.281 **	1.000												
well-being	−0.048 **	−0.082 **	−0.082 **	−0.034 **	0.046 **	−0.046 **	0.054 **	0.075 **	0.212 **	1.000											
migrant	0.081 **	−0.178 **	−0.180 **	−0.001	0.102 **	−0.065 **	0.013	0.138 **	0.002	0.037 **	1.000										
lnphi	0.254 **	−0.705 **	−0.711 **	0.095 **	0.347 **	−0.002	0.127 **	0.428 **	0.210 **	0.144 **	0.211 **	1.000									
lnPGDP	0.259 **	−0.365 **	−0.371 **	0.086 **	0.159 **	−0.009	0.091 **	0.280 **	0.091 **	0.016	0.145 **	0.444 **	1.000								
lnPGDP2	0.260 **	−0.367 **	−0.373 **	0.087 **	0.160 **	−0.008	0.092 **	0.281 **	0.091 **	0.016	0.146 **	0.447 **	1.000 **	1.000							
pop den	−0.044 **	0.099 **	0.104 **	0.021	−0.018	0.008	−0.059 **	−0.061 **	−0.075 **	−0.046 **	−0.072 **	−0.108 **	−0.346 **	−0.344 **	1.000						
pm25	0.103 **	−0.052 **	−0.052 **	0.026 *	0.024 *	−0.038 **	0.063 **	0.021	0.068 **	−0.030 **	−0.058 **	0.096 **	0.333 **	0.335 **	−0.168 **	1.000					
garbage	0.022	−0.112 **	−0.109 **	0.024 *	0.073 **	−0.011	0.005	0.089 **	0.063 **	0.039 **	0.050 **	0.123 **	0.317 **	0.309 **	−0.165 **	0.178 **	1.000				
nox	−0.120 **	0.184 **	0.189 **	−0.065 **	−0.009	−0.035 **	−0.019	−0.125 **	0.059 **	0.046 **	−0.102 **	−0.181 **	−0.245 **	−0.254 **	−0.003	0.199 **	0.508 **	1.000			
so2	−0.161 **	0.262 **	0.268 **	−0.081 **	−0.052 **	−0.027 *	−0.028 *	−0.213 **	0.035 **	0.042 **	−0.114 **	−0.278 **	−0.476 **	−0.484 **	−0.004	0.077 **	0.316 **	0.907 **	1.000		
wat poll	−0.073 **	−0.015	−0.011	0.013	0.010	−0.018	0.012	−0.018	0.057 **	0.029 *	−0.026 *	0.010	0.125 **	0.117 **	−0.190 **	0.345 **	0.879 **	0.641 **	0.485 **	1.000	
smoke	−0.182 **	0.299 **	0.304 **	−0.107 **	−0.076 **	−0.026 *	−0.059 **	−0.175 **	0.026 *	0.041 **	−0.111 **	−0.321 **	−0.519 **	−0.529 **	0.068 **	−0.127 **	0.248 **	0.868 **	0.878 **	0.359 **	1.000

Note: Asterisks indicate the statistical significance, where * and ** denote significance at 10% and 5% levels, respectively

**Table A2:** Akaike’s and Schwarz’s Bayesian information criteria.

Model	Model 1		Model 2		Model 3	
	AIC	BIC	AIC	BIC	AIC	BIC
OLS	24,261.9	24,282.59	22,229.68	22,311.62	21,916.78	22,060.18
OLM	46,060.09	46,246.32	42,537.73	42,783.55	42,219.33	42,526.6

Note: Model 1 denotes the model that only includes individual income inequality and its square. Model 2 is the model that controls individual characteristics based on Model 1. Model 3 further controls the effect of city factors based on Model 2.

**Table A3:** Ramsey RESET test.

Model	Model 1	Model 2	Model 3
F	22.44	5.23	4.84
*p*-value	0.0000	0.0013	0.0023

Note: Model 1 denotes the model that only includes individual income inequality and its square. Model 2 is the model that controls individual characteristics based on Model 1. Model 3 further controls the effect of city factors based on Model 2.

**Table A4:** Mean of individual-level income inequality and subjective environmental pollution for the respondents that use different media and have different use frequency.

Variables	Frequency	Never	Rarely	Sometimes	Often	Very Often
newspaper	RD	9.56	9.22	9.07	8.93	8.75
	SEP	2.58	2.93	3.16	3.14	2.95
magazine	RD	9.48	9.15	9.02	8.94	8.67
	SEP	2.68	2.99	3.11	3.04	2.9
broadcasting	RD	9.38	9.19	9.09	9.1	8.94
	SEP	2.71	2.95	3.11	3.16	2.92
television	RD	9.33	9.18	9.2	9.26	9.26
	SEP	2.81	2.92	2.91	2.91	2.82
internet(including mobile phone	RD	9.52	9.17	9.08	8.97	8.8
	SEP	2.65	3.04	3.03	3.1	3.19
mobile custom message	RD	9.39	9.05	8.94	8.97	8.78
	SEP	2.76	3.05	3.11	3.16	3.16

**Table A5:** Variable description.

Variables	Description
Air pollution	Subjective air pollution: 0 = this problem does not exist, 1 = indifferent, 2 = not serious, 3 = very serious, 4 = somewhat serious, and 5 = very serious
Water pollution	Subjective water pollution: 0 = this problem does not exist, 1 = indifferent, 2 = not serious, 3 = very serious, 4 = somewhat serious, and 5 = very serious
Noise pollution	Subjective noise pollution: 0 = this problem does not exist, 1 = indifferent, 2 = not serious, 3 = very serious, 4 = somewhat serious, and 5 = very serious
Industrial waste pollution	Subjective industrial waste pollution: 0 = this problem does not exist, 1 = indifferent, 2 = not serious, 3 = very serious, 4 = somewhat serious, and 5 = very serious
Garbage pollution	Subjective garbage pollution: 0 = this problem does not exist, 1 = indifferent, 2 = not serious, 3 = very serious, 4 = somewhat serious, and 5 = very serious
SEP	Subjective environmental pollution
$Yitzhaki_{in_{i}r_{a}}$	Yitzhaki index, which is calculated by the personal income of respondents in full samples
$Yitzhaki_{in_{i}r_{s}}$	Yitzhaki index, which is calculated by the personal income of respondents that have non-zero income
$Yitzhaki_{in_{h}r_{s}}$	Yitzhaki index, which is calculated by the total household income of respondents that have non-zero income
$Yitzhaki_{in_{p}r_{s}}$	Yitzhaki index, which is calculated by the household income per capita of respondents that have non-zero income
${Kakwani}_{{in}_{i}r_{a}}$	Kakwani index, which is calculated by the personal income of respondents in full samples
${Kakwani}_{{in}_{i}r_{s}}$	Kakwani index, which is calculated by the personal income of respondents that have non-zero income
${Kakwani}_{{in}_{h}r_{s}}$	Kakwani index, which is calculated by the total household income of respondents that have non-zero income
${Kakwani}_{{in}_{p}r_{s}}$	Kakwani index, which is calculated by the household income per capita of respondents that have non-zero income
env behavior	the behavior of environment
env know	the knowledge of environment
gender	male = 1 and female = 0
env attitude	environmental attitude
ind cog	cognitive level
health	1—very unhealthy; 2—relatively unhealthy; 3—general; 4—relatively healthy; 5—very healthy
well-being	1—very unhappy; 2—less happy; 3—cannot say happiness or unhappiness; 4—comparatively happy; 5—very happy
migrant	migrant = 1 and the local = 0
lnphi	the logarithm of household income per capita
lnPGDP	the logarithm of GDP per capita
lnPGDP2	the square of lnPGDP
pop den	the logarithm population density
pm_2.5_	the logarithm of the concentration of PM_2.5_
garbage	the logarithm of the amount of municipal solid waste
nox	the logarithm of total emission of NOx
so2	the logarithm of total emission of SO_2_
wat poll	the logarithm of total discharge of waste water
smo	the logarithm of total emission of smoke (dust)

**Table A6:** The effect of individual-level income inequality on different subjective environmental pollution.

	(1)	(2)	(3)	(4)	(5)
	Air pollution	Water pollution	Noise pollution	Industrial waste pollution	Garbage pollution
inequality	1.252 ***	0.945 **	1.973 ***	0.752 *	0.819 *
	(2.79)	(2.32)	(4.77)	(1.71)	(1.90)
inequality^2^	−0.074 ***	−0.056 **	−0.118 ***	−0.044 *	−0.043 *
	(−2.86)	(−2.40)	(−4.95)	(−1.75)	(−1.72)
lnphi	0.170 ***	0.072 **	0.155 ***	0.108 ***	0.127 ***
	(4.85)	(2.08)	(4.41)	(3.09)	(3.63)
env behavior	0.282 ***	0.381 ***	0.286 ***	0.385 ***	0.266 ***
	(4.24)	(5.68)	(4.30)	(5.92)	(4.19)
env know	0.057 ***	0.069 ***	0.066 ***	0.052 ***	0.051 ***
	(6.11)	(7.32)	(6.98)	(5.54)	(5.37)
gender	−0.077 *	−0.082 *	−0.114 **	−0.030	−0.030
	(−1.65)	(−1.74)	(−2.43)	(−0.63)	(−0.64)
env attitude	0.142 ***	0.107 ***	0.055 *	0.040	0.020
	(4.65)	(3.44)	(1.86)	(1.29)	(0.67)
ind cong	0.232 ***	0.138 ***	0.224 ***	0.192 ***	0.204 ***
	(6.28)	(3.70)	(6.13)	(5.11)	(5.58)
health	−0.053 **	−0.014	−0.031	0.051 **	0.012
	(−2.32)	(−0.60)	(−1.32)	(2.18)	(0.51)
well-being	−0.122 ***	−0.121 ***	−0.133 ***	−0.171 ***	−0.126 ***
	(−4.10)	(−4.17)	(−4.45)	(−5.84)	(−4.37)
lnPGDP	−18.857 ***	−1.048	3.388	3.335	1.970
	(−4.32)	(−0.25)	(0.77)	(0.92)	(0.52)
lnPGDP2	0.914 ***	0.053	−0.124	−0.121	−0.076
	(4.50)	(0.27)	(−0.61)	(−0.73)	(−0.43)
pop den	0.058	−0.095	0.156 **	0.268 ***	0.111 *
	(0.95)	(−1.53)	(2.46)	(4.34)	(1.83)
pm25	0.587 ***	0.427 ***	0.274 ***	0.319 ***	−0.040
	(8.90)	(6.33)	(3.99)	(5.60)	(−0.70)
garbage	0.562 ***	0.701 ***	0.921 ***	−0.072	−0.271 ***
	(5.82)	(7.41)	(9.52)	(−1.45)	(−5.34)
nox	−0.374 **	−0.036	−0.525 ***	−0.557 ***	−0.009
	(−2.26)	(−0.21)	(−3.00)	(−4.70)	(−0.08)
so2	0.214 **	0.185 *	0.633 ***	0.422 ***	0.074
	(2.14)	(1.81)	(6.30)	(4.20)	(0.77)
wat poll	−0.679 ***	−0.665 ***	−1.086 ***		
	(−7.00)	(−6.87)	(−10.98)		
smoke	0.224 *	−0.201 *	−0.019		
	(1.88)	(−1.74)	(−0.16)		
N	6825	6825	6825	6825	6825

Notes: The parentheses show *t* values of robust standard error. Asterisks indicate the statistical significance, where *, **, and *** denote significance at 10%, 5%, and 1% levels, respectively. The results are estimated by an ordered logit model.

**Table A7:** The effect of individual-level income inequality on subjective environmental pollution: robustness test based on different measurements of income inequality at the individual level.

	(1)	(2)	(3)	(4)	(5)	(6)	(7)
	$Yitzhaki_{in_{i}r_{s}}$	$Yitzhaki_{in_{h}r_{s}}$	$Yitzhaki_{in_{p}r_{s}}$	${\mathrm{Kakwani}}_{{in}_{i}r_{a}}$	${Kakwani}_{{in}_{i}r_{s}}$	${Kakwani}_{{in}_{hi}r_{s}}$	${Kakwani}_{{in}_{p}r_{s}}$
inequality	1.784 ***	1.835 **	6.231 ***	0.435	1.400 ***	1.119 ***	0.121
	(3.84)	(2.08)	(4.38)	(1.13)	(3.35)	(2.59)	(0.21)
inequality^2^	−0.103 ***	−0.086 *	−0.362 ***	−0.597 *	−1.780 ***	−0.910 **	−1.601 ***
	(−3.84)	(−1.86)	(−4.27)	(−1.91)	(−4.75)	(−2.34)	(−3.63)
lnphi	0.203 ***	0.252 ***	0.047	0.150 ***	0.155 ***	0.218 ***	−0.166 *
	(5.28)	(5.41)	(0.55)	(4.43)	(4.03)	(4.55)	(−1.77)
env behavior	0.433 ***	0.457 ***	0.452 ***	0.452 ***	0.440 ***	0.459 ***	0.454 ***
	(6.14)	(6.72)	(6.65)	(6.63)	(6.28)	(6.74)	(6.67)
env know	0.067 ***	0.071 ***	0.069 ***	0.070 ***	0.067 ***	0.072 ***	0.069 ***
	(6.84)	(7.61)	(7.39)	(7.54)	(6.78)	(7.66)	(7.42)
gender	−0.047	−0.052	−0.051	−0.087 *	−0.056	−0.052	−0.050
	(−0.96)	(−1.21)	(−1.18)	(−1.87)	(−1.16)	(−1.20)	(−1.16)
env attitude	0.082 ***	0.090 ***	0.090 ***	0.087 ***	0.082 ***	0.088 ***	0.091 ***
	(2.77)	(3.18)	(3.21)	(3.07)	(2.76)	(3.12)	(3.23)
ind cong	0.203 ***	0.232 ***	0.234 ***	0.223 ***	0.202 ***	0.228 ***	0.232 ***
	(5.13)	(6.26)	(6.27)	(5.97)	(5.10)	(6.17)	(6.24)
health	−0.007	−0.003	−0.006	−0.007	−0.018	−0.004	−0.006
	(−0.31)	(−0.14)	(−0.25)	(−0.29)	(−0.75)	(−0.16)	(−0.25)
well-being	−0.178 ***	−0.162 ***	−0.167 ***	−0.165 ***	−0.174 ***	−0.163 ***	−0.166 ***
	(−5.93)	(−5.63)	(−5.83)	(−5.75)	(−5.80)	(−5.66)	(−5.81)
lnPGDP	−4.421	−6.640	−5.724	−6.435	−4.540	−7.163 *	−5.766
	(−1.00)	(−1.56)	(−1.34)	(−1.51)	(−1.02)	(−1.68)	(−1.36)
lnPGDP2	0.228	0.334 *	0.291	0.325	0.233	0.359 *	0.293
	(1.10)	(1.69)	(1.47)	(1.64)	(1.12)	(1.81)	(1.48)
pop den	0.014	0.041	0.056	0.052	0.000	0.037	0.053
	(0.22)	(0.67)	(0.92)	(0.85)	(0.01)	(0.60)	(0.87)
pm25	0.556 ***	0.569 ***	0.557 ***	0.586 ***	0.565 ***	0.573 ***	0.559 ***
	(8.02)	(8.48)	(8.37)	(8.81)	(8.15)	(8.55)	(8.41)
garbage	0.837 ***	0.831 ***	0.843 ***	0.836 ***	0.845 ***	0.830 ***	0.847 ***
	(8.54)	(8.76)	(8.90)	(8.83)	(8.65)	(8.75)	(8.93)
nox	−0.450 **	−0.459 ***	−0.426 **	−0.474 ***	−0.421 **	−0.472 ***	−0.428 **
	(−2.55)	(−2.73)	(−2.54)	(−2.82)	(−2.38)	(−2.80)	(−2.55)
so2	0.323 ***	0.298 ***	0.299 ***	0.305 ***	0.308 ***	0.296 ***	0.298 ***
	(3.11)	(2.98)	(3.00)	(3.05)	(2.95)	(2.96)	(2.98)
wat poll	−0.966 ***	−0.982 ***	−0.994 ***	−0.994 ***	−0.968 ***	−0.984 ***	−0.997 ***
	(−9.72)	(−10.28)	(−10.42)	(−10.44)	(−9.77)	(−10.31)	(−10.46)
smoke	0.102	0.175	0.145	0.197	0.084	0.192	0.147
	(0.81)	(1.45)	(1.20)	(1.64)	(0.67)	(1.60)	(1.22)
N	6211	6825	6825	6826	6212	6826	6826

Notes: Asterisks indicate the statistical significance, where ***, **, and * represent significance at 1%, 5%, and 10% levels. The parentheses show *t* values of robust standard error. The results in this table are estimated by an ordered logit model.

**Table A8:** The effect of individual-level income inequality on subjective environmental pollution: robustness test based on cluster analysis and an ordered probit model.

	(1)	(2)	(3)	(4)
	clustered at province level	clustered at city level	clustered at county level	probit
inequality	1.317 **	1.317 **	1.317 **	0.737 ***
	(2.39)	(2.46)	(2.53)	(3.11)
inequality^2^	−0.077 **	−0.077 **	−0.077 ***	−0.043 ***
	(−2.36)	(−2.41)	(−2.59)	(−3.17)
lnphi	0.165 ***	0.165 ***	0.165 ***	0.088 ***
	(3.95)	(3.47)	(3.40)	(4.59)
env behavior	0.451 ***	0.451 ***	0.451 ***	0.248 ***
	(3.35)	(3.39)	(4.33)	(6.70)
env know	0.070 ***	0.070 ***	0.070 ***	0.042 ***
	(4.01)	(3.83)	(4.25)	(7.85)
gender	−0.078 *	−0.078	−0.078	−0.046 *
	(−1.67)	(−1.40)	(−1.61)	(−1.73)
env attitude	0.087 **	0.087 *	0.087 **	0.052 ***
	(2.26)	(1.94)	(2.04)	(3.29)
ind cong	0.223 ***	0.223 ***	0.223 ***	0.132 ***
	(3.34)	(3.05)	(3.63)	(6.28)
health	−0.005	−0.005	−0.005	−0.003
	(−0.13)	(−0.15)	(−0.16)	(−0.25)
well-being	−0.168 ***	−0.168 ***	−0.168 ***	−0.093 ***
	(−2.98)	(−3.10)	(−4.30)	(−5.73)
lnPGDP	−6.250	−6.250	−6.250	−4.794 *
	(−0.29)	(−0.34)	(−0.40)	(−1.95)
lnPGDP2	0.316	0.316	0.316	0.239 **
	(0.31)	(0.37)	(0.43)	(2.09)
pop den	0.055	0.055	0.055	0.031
	(0.22)	(0.23)	(0.25)	(0.88)
pm25	0.573 *	0.573 **	0.573 ***	0.334 ***
	(1.77)	(1.98)	(2.61)	(8.74)
garbage	0.833 **	0.833 **	0.833 **	0.458 ***
	(2.08)	(2.23)	(2.47)	(8.37)
nox	−0.463	−0.463	−0.463	−0.286 ***
	(−0.54)	(−0.63)	(−0.76)	(−2.99)
so2	0.305	0.305	0.305	0.197 ***
	(0.68)	(0.76)	(0.85)	(3.51)
wat poll	−0.990 **	−0.990 ***	−0.990 ***	−0.547 ***
	(−2.25)	(−2.66)	(−2.94)	(−9.98)
smoke	0.186	0.186	0.186	0.120 *
	(0.30)	(0.34)	(0.41)	(1.80)
N	6825	6825	6825	6825

Note: The parentheses show *t* statistics, where columns 1–3 are *t* values of clustering robust standard error. Asterisks indicate the statistical significance, where ***, **, and * represent significance at 1%, 5%, and 10% levels, respectively. The column 1 clusters are at the province level, column 2 clusters are at the city level, column 3 clusters are at the county level. Column 4 is estimated by an ordered probit model.

**Table A9:** The effect of individual-level income inequality on subjective environmental pollution: differences in urban and rural area.

	(1)	(2)	(3)	(4)	(5)	(6)
	The urban area			The rural area		
inequality	0.982 **	1.219 ***	0.931 **	−1.029	−1.330	−1.329
	(2.43)	(2.93)	(2.24)	(−1.27)	(−1.58)	(−1.31)
inequality^2^	−0.069 ***	−0.071 ***	−0.053 **	0.040	0.067	0.068
	(−2.94)	(−2.90)	(−2.18)	(0.90)	(1.41)	(1.20)
lnphi		0.133 ***	0.049		0.076	0.041
		(2.79)	(1.01)		(1.49)	(0.79)
env behavior		0.495 ***	0.471 ***		0.089	0.141
		(6.80)	(6.41)		(0.70)	(1.09)
env know		0.007	0.014		0.110 ***	0.112 ***
		(0.63)	(1.19)		(6.93)	(7.03)
gender		−0.018	−0.007		−0.166 **	−0.137 *
		(−0.32)	(−0.13)		(−2.06)	(−1.70)
env attitude		0.058 *	0.046		0.217 ***	0.223 ***
		(1.72)	(1.34)		(4.37)	(4.42)
ind cong		0.290 ***	0.260 ***		0.025	0.062
		(6.65)	(5.96)		(0.37)	(0.89)
health		0.000	−0.000		0.029	0.019
		(0.01)	(−0.01)		(0.83)	(0.54)
well-being		−0.232 ***	−0.217 ***		−0.020	−0.024
		(−6.42)	(−5.97)		(−0.44)	(−0.53)
lnPGDP			0.495 ***			−19.900 ***
			(6.77)			(−2.60)
lnPGDP2						0.964 ***
						(2.68)
pop den			0.069			0.187 *
			(1.01)			(1.85)
pm25			0.076			0.211 ***
			(1.20)			(2.61)
N	4761	4457	4457	2550	2368	2368

Notes: The parentheses show *t* value of robust standard error. Asterisks indicate the statistical significance, where *, **, and *** denote significance at 10%, 5%, and 1% levels, respectively. The results in this table are estimated by an ordered logit model.

**Table A10:** The effect of individual-level income inequality on subjective environmental pollution: differences between the locals and migrants.

	(1)	(2)	(3)	(4)	(5)	(6)
	Local			Migrant		
inequality	2.127 ***	2.004 ***	1.407 **	1.312 **	0.943	0.393
	(4.40)	(3.80)	(2.49)	(2.05)	(1.44)	(0.63)
inequality^2^	−0.152 ***	−0.124 ***	−0.087 ***	−0.094 **	−0.048	−0.015
	(−5.55)	(−4.12)	(−2.71)	(−2.54)	(−1.25)	(−0.40)
lnphi		0.227 ***	0.127 ***		0.308 ***	0.222 ***
		(5.79)	(3.25)		(4.48)	(3.19)
env behavior		0.426 ***	0.428 ***		0.457 ***	0.471 ***
		(5.07)	(4.93)		(4.66)	(4.42)
env know		0.079 ***	0.080 ***		0.026	0.044 **
		(7.10)	(7.20)		(1.48)	(2.56)
gender		−0.229 ***	−0.176 ***		0.243 ***	0.226 **
		(−4.18)	(−3.22)		(2.73)	(2.54)
env attitude		0.108 ***	0.095 ***		0.087 *	0.101 **
		(3.31)	(2.81)		(1.75)	(1.99)
ind cong		0.351 ***	0.298 ***		0.105	0.055
		(7.89)	(6.54)		(1.60)	(0.83)
health		−0.072 ***	−0.061 **		0.143 ***	0.133 ***
		(−2.70)	(−2.24)		(3.41)	(3.10)
well-being		−0.210 ***	−0.191 ***		−0.157 ***	−0.112 **
		(−6.21)	(−5.55)		(−3.05)	(−2.11)
lnPGDP			−10.552 **			1.126
			(−2.10)			(0.13)
lnPGDP2			0.516 **			−0.023
			(2.21)			(−0.06)
pop den			0.078			0.006
			(1.08)			(0.04)
pm25			0.477 ***			0.905 ***
			(6.20)			(6.14)
garbage			0.893 ***			0.544 ***
			(7.78)			(2.83)
nox			−0.377 *			−0.301
			(−1.93)			(−0.81)
so2			0.309 ***			0.192
			(2.69)			(0.91)
wat poll			−0.931 ***			−1.109 ***
			(−8.36)			(−5.58)
smoke			0.135			0.184
			(0.94)			(0.75)
N	5241	4885	4885	2069	1940	1940

Notes: The parentheses show *t* values of robust standard error. Asterisks indicates the statistical significance, where *, **, and *** denote significance at 10%, 5%, and 1% levels, respectively. The results in this table are estimated by ordered logit model.

**Table A11:** The effect of individual-level income inequality on subjective environmental pollution: differences in gender.

	(1)	(2)	(3)	(4)	(5)	(6)
	The female group			The male group		
inequality	2.958 ***	2.700 ***	1.977 **	2.023 ***	1.763 ***	1.237 ***
	(3.71)	(3.02)	(2.26)	(4.48)	(3.87)	(2.59)
inequality^2^	−0.195 ***	−0.155 ***	−0.111 **	−0.148 ***	−0.106 ***	−0.074 ***
	(−4.40)	(−3.13)	(−2.28)	(−5.64)	(−3.92)	(−2.61)
lnphi		0.217 ***	0.135 ***		0.306 ***	0.198 ***
		(4.67)	(2.96)		(6.22)	(3.90)
env behavior		0.649 ***	0.625 ***		0.285 ***	0.318 ***
		(6.55)	(5.98)		(3.38)	(3.64)
env know		0.070 ***	0.072 ***		0.056 ***	0.067 ***
		(5.00)	(5.23)		(4.44)	(5.32)
env attitude		0.128 ***	0.124 ***		0.058	0.051
		(3.34)	(3.13)		(1.50)	(1.28)
ind cong		0.236 ***	0.207 ***		0.338 ***	0.251 ***
		(4.41)	(3.86)		(6.64)	(4.79)
health		−0.025	−0.028		0.003	0.011
		(−0.77)	(−0.84)		(0.10)	(0.35)
wellbeing		−0.161 ***	−0.134 ***		−0.226 ***	−0.199 ***
		(−3.85)	(−3.17)		(−5.89)	(−5.08)
lnPGDP			−7.381			−5.540
			(−1.16)			(−0.96)
lnPGDP2			0.379			0.275
			(1.28)			(1.02)
pop den			0.155 *			−0.018
			(1.68)			(−0.23)
pm25			0.531 ***			0.591 ***
			(5.25)			(6.73)
garbage			0.713 ***			0.921 ***
			(5.02)			(7.18)
nox			−0.645 ***			−0.339
			(−2.59)			(−1.49)
so2			0.402 ***			0.238 *
			(2.64)			(1.80)
wat poll			−0.942 ***			−1.008 ***
			(−6.61)			(−7.79)
smoke			0.336 *			0.066
			(1.90)			(0.40)
N	3372	3124	3124	3942	3704	3704

Notes: The parentheses show *t* values of robust standard error. Asterisks indicate the statistical significance, where *, **, and *** denote significance at 10%, 5%, and 1% levels, respectively.

**Table A12:** The effect of individual-level income inequality on subjective environmental pollution: differences in environmental attitude.

	(1)	(2)	(3)	(4)	(5)	(6)
	Negative attitude group			Positive attitude group		
inequality	1.872 ***	1.588 ***	0.858 *	2.312 ***	2.447 ***	2.143 ***
	(3.90)	(3.10)	(1.67)	(3.51)	(3.62)	(3.11)
inequality^2^	−0.134 ***	−0.095 ***	−0.051 *	−0.158 ***	−0.146 ***	−0.127 ***
	(−4.95)	(−3.26)	(−1.73)	(−4.07)	(−3.61)	(−3.06)
lnphi		0.269 ***	0.177 ***		0.209 **	0.097
		(7.30)	(4.81)		(2.42)	(1.14)
env behavior		0.438 ***	0.441 ***		0.411 ***	0.411 ***
		(5.91)	(5.78)		(2.86)	(2.73)
env know		0.064 ***	0.069 ***		0.049 *	0.067 **
		(6.39)	(6.90)		(1.96)	(2.56)
gender		−0.111 **	−0.072		−0.159	−0.137
		(−2.16)	(−1.41)		(−1.44)	(−1.23)
ind cong		0.285 ***	0.232 ***		0.273 ***	0.220 **
		(6.97)	(5.63)		(3.26)	(2.56)
health		−0.011	−0.001		−0.018	−0.043
		(−0.44)	(−0.06)		(−0.31)	(−0.70)
well-being		−0.186 ***	−0.166 ***		−0.246 ***	−0.202 ***
		(−6.07)	(−5.34)		(−3.39)	(−2.72)
lnPGDP			−1.561			−12.847
			(−0.42)			(−1.23)
lnPGDP2			0.100			0.602
			(0.58)			(1.24)
pop den			0.053			0.022
			(0.80)			(0.14)
pm25			0.529 ***			0.720 ***
			(7.86)			(3.96)
garbage			0.790 ***			0.963 ***
			(7.54)			(4.16)
nox			−0.294 **			−0.360
			(−2.24)			(−0.81)
so2			0.343 ***			0.111
			(3.21)			(0.40)
wat poll			−1.059 ***			−0.820 ***
			(−10.49)			(−3.45)
smoke						0.227
						(0.79)
N	6105	5696	5696	1209	1132	1132

Notes: The parentheses show *t* values of robust standard error. Asterisks indicate the statistical significance, where *, **, and *** denote significance at 10%, 5%, and 1% levels, respectively.

**Table A13:** The mechanism of the effect of individual-level income inequality on subjective environmental pollution.

	(1)	(2)	(3)	(4)	(5)	(6)
	newspaper	magazine	broadcasting	television	Internet (including surfing the Internet with mobile phone)	mobile custom messages
inequality	0.590 **	0.631 **	0.875 ***	0.900 ***	0.457	0.610 **
	(2.01)	(2.23)	(2.97)	(3.28)	(1.48)	(2.22)
inequality^2^	−0.039 **	−0.043 ***	−0.054 ***	−0.052 ***	−0.034 **	−0.042 ***
	(−2.42)	(−2.73)	(−3.27)	(−3.31)	(−2.03)	(−2.70)
media	−0.234	−0.371 **	−0.230	0.004	−0.308 **	−0.407 ***
	(−1.48)	(−2.15)	(−1.42)	(0.02)	(−2.33)	(−2.86)
inequality∗media	0.029 *	0.042 **	0.030 *	−0.002	0.036 **	0.049 ***
	(1.68)	(2.18)	(1.68)	(−0.09)	(2.50)	(3.07)
health	0.005	0.002	0.009	0.005	−0.002	−0.001
	(0.29)	(0.14)	(0.54)	(0.29)	(−0.10)	(−0.04)
well-being	−0.091 ***	−0.090 ***	−0.092 ***	−0.089 ***	−0.089 ***	−0.091 ***
	(−4.72)	(−4.68)	(−4.73)	(−4.58)	(−4.61)	(−4.69)
env know	0.040 ***	0.041 ***	0.044 ***	0.042 ***	0.039 ***	0.041 ***
	(6.11)	(6.28)	(6.66)	(6.42)	(5.90)	(6.23)
lnphi	0.101 ***	0.104 ***	0.107 ***	0.109 ***	0.096 ***	0.101 ***
	(4.10)	(4.21)	(4.27)	(4.41)	(3.85)	(4.11)
env behavior	0.230 ***	0.234 ***	0.197 ***	0.234 ***	0.231 ***	0.224 ***
	(5.67)	(5.77)	(4.87)	(5.75)	(5.72)	(5.51)
gender	−0.061 *	−0.056 *	−0.067 **	−0.053	−0.054	−0.057 *
	(−1.87)	(−1.70)	(−2.01)	(−1.61)	(−1.63)	(−1.73)
env attitude	0.046 **	0.046 **	0.043 **	0.045 **	0.046 **	0.045 **
	(2.47)	(2.47)	(2.33)	(2.42)	(2.50)	(2.42)
ind cong	0.101 ***	0.106 ***	0.128 ***	0.111 ***	0.088 ***	0.096 ***
	(3.97)	(4.15)	(5.09)	(4.43)	(3.21)	(3.71)
migrant	−0.002	−0.002	0.004	−0.000	−0.000	−0.001
	(−0.07)	(−0.07)	(0.12)	(−0.01)	(−0.01)	(−0.04)
lnPGDP	0.038	−0.133	−1.348	−0.180	−0.440	−0.532
	(0.01)	(−0.05)	(−0.53)	(−0.07)	(−0.17)	(−0.21)
lnPGDP2	0.016	0.024	0.087	0.027	0.038	0.042
	(0.13)	(0.20)	(0.74)	(0.22)	(0.32)	(0.36)
pop den	0.015	0.021	0.121 ***	0.022	0.020	0.017
	(0.36)	(0.50)	(2.84)	(0.52)	(0.46)	(0.41)
pm25	0.349 ***	0.345 ***	0.114 ***	0.346 ***	0.344 ***	0.347 ***
	(7.51)	(7.42)	(2.75)	(7.43)	(7.42)	(7.48)
garbage	0.548 ***	0.536 ***		0.536 ***	0.546 ***	0.544 ***
	(8.44)	(8.25)		(8.21)	(8.39)	(8.36)
nox	−0.261 ***	−0.272 ***	−0.557 ***	−0.286 ***	−0.265 ***	−0.255 ***
	(−2.88)	(−3.00)	(−6.76)	(−3.16)	(−2.91)	(−2.81)
so2	0.375 ***	0.381 ***	0.461 ***	0.392 ***	0.374 ***	0.365 ***
	(5.19)	(5.27)	(6.45)	(5.43)	(5.16)	(5.05)
wat poll	−0.761 ***	−0.746 ***		−0.746 ***	−0.754 ***	−0.754 ***
	(−11.72)	(−11.48)		(−11.44)	(−11.58)	(−11.60)
N	6824	6822	6817	6819	6814	6818
R^2^	0.117	0.117	0.099	0.116	0.117	0.118

Notes: The parentheses show *t* values of robust standard error. Asterisks indicate the statistical significance, where *, **, and *** denote significance at 10%, 5%, and 1% levels, respectively. The results in this table are estimated by OLS.
